# Fearless and Independent

**Published:** 1904-10

**Authors:** 


### Fearless and Independent.

The Chicago Record-Herald is a conspicuous example of the success with which the public rewards fearless non-partisanship in the columns of a great metropolitan daily paper. It is an independent newspaper, in which men and measures are invariably viewed wholly from the standpoint of the public good and not from that of the interests of any particular political party. It is the very reverse of neutral—fearless and outspoken on all the great questions of the day, but presenting its editorial opinion upon independent judgment and entirely regardless of political affiliations. Partisanship is barred as strictly from the news columns as from the editorial page. All political news is given without partisan coloring, thus enabling the reader to form correct conclusions for himself. In the ordinary partisan newspaper political news is usually colored to such an extent as to make it difficult if not impossible for the reader to secure a sound basis for intelligent judgment.

